# The power of collective voices

**DOI:** 10.1093/eurpub/ckae111

**Published:** 2024-08-01

**Authors:** Charlotte Marchandise, Hans Henri P Kluge, Ricardo Mexia, Floris Barnhoorn

**Affiliations:** EUPHA Executive Director; WHO Regional Director for Europe, By Hans Henri P. Kluge Gundo Weiler, Marie Wolf; Chair of the 17th EPH Conference, Lisbon 2024; Deputy Director EPH Conference

Dear readers,

The European Public Health Association has always been a beacon of collaboration, innovation, and impactful action in the realm of public health. As we are creating our new strategy for 2025–2030, it is with immense pride and gratitude that we reflect on our recent strategic retreat, which marked a significant milestone in our journey. This retreat was not just a meeting of minds but a celebration of our collective commitment to advancing public health across Europe and beyond.

Our strategic retreat was shaped by the voices of our members. Over the past few months, we conducted extensive surveys across our diverse sections and membership base. The response was overwhelming, with a rich tapestry of insights, suggestions, and feedback from all the diversity of our community. These inputs were instrumental in guiding our discussions and shaping our strategic direction.

The engagement from our members underscored a shared vision for EUPHA—one that is rooted in our 30-year legacy of remarkable achievements and driven by a collective aspiration for transformative impact. The active participation of our staff, executive council, members of the governing board, and representatives from our 29 thematic sections ensured that our strategy is reflective of the broad spectrum of expertise and perspectives within EUPHA.

Amongst the many elements shared, which we will formally present when we finish the analysis, what was very clear is the essence of EUPHA is about building bridges for transformative impact. We aim to create a connected, dynamic, and empowered community that not only drives positive change but also fosters continuous learning. This vision is underpinned by a mission to engage diverse stakeholders through outreach and empowerment, sharing a vision, and inspiring meaningful impact.

We also see a thirst for innovation and new ways of connecting and working together. We have been working with a team of designer, to build our new tools based on the uses of all our community, and we will renew our digital tools in the coming months.

Your voice, your ideas, and your dedication are what make EUPHA a force.

Let’s build bridges together for health, equity, inclusiveness and sustainability.

## Health for all? For many migrants and refugees in the WHO European Region that vision remains far from reality

KlugeHans Henri P
WHO Regional Director for Europe, By Hans Henri P. Kluge Gundo Weiler, Marie Wolf

EJPH

People have always been on the move. It is how societies and cultures are built. In our globalized world of today, the scale and impact of migration are increasing. While in 1990 ∼150 million people lived outside their country of birth, this number had risen to 281 million in 2022 (https://worldmigrationreport.iom.int/msite/wmr-2024-interactive/). And of the six WHO Regions, it is the European Region that currently hosts the largest number of international migrants—101 million—or 36% of the global migrant population (UN DESA, Migrant stock data, 2020 https://www.un.org/development/desa/pd/content/international-migrant-stock).

Social factors play an important role in determining the health outcomes of different population groups (https://www.who.int/initiatives/action-on-the-social-determinants-of-health-for-advancing-equity/world-report-on-social-determinants-of-health-equity/commission-on-social-determinants-of-health). Migration and displacement are key social determinants of health.

Generally, refugees and migrants across Europe and Central Asia have the same health needs as host populations. But they face heightened risks of ill health, both during transit and in destination countries, stemming from a combination of factors including dangerous migration routes, poor living conditions, and changes in lifestyle.

Restrictive migration policies, issues related to legal status, economic hardship, and anti-migrant sentiment—all impact the health and well-being of refugees and migrants.

As a result, refugees and migrants often face severe challenges in accessing care—in their countries of origin, during transit and in host countries. When navigating new health systems, their experiences can be very different to those of host communities. Refugees and migrants might not enjoy entitlements to health services beyond immediate emergency care—and even that can be challenging. They often also face language barriers or struggle with out-of-pocket costs.

Recognizing these challenges and the importance of addressing both immediate and long-term needs, the 53 Member States of the European Region, in a milestone agreement, adopted a new action plan last year (Action plan for refugee and migrant health in the WHO European Region 2023–2030. https://www.who.int/europe/publications/i/item/WHO-EURO-2023-8966-48738-72475), outlining concrete action aimed at improving refugee and migrant health through 2030.

First, WHO Member States across Europe and Central Asia called for the inclusion of refugees and migrants in universal health coverage. While this is essential to fulfilling WHOs vision of Health For All, its implementation remains elusive. The right to health specifies that every individual should be able to enjoy the highest attainable standard of health. However, for this to become reality, for us to respond to the immediate and longer-term health needs of refugees and migrants, public health systems must be strengthened significantly to accommodate these needs.

Second, Member States agreed to make emergency and disaster risk-reduction policies and actions truly inclusive. This means refugees and migrants are to be accounted for in mitigation, preparedness, response, and recovery processes, including in protracted situations resulting from conflicts, natural disasters, or other crises. During emergencies and disasters, refugees and migrants are often denied access to necessary resources and services. At the same time, they can be more vulnerable as the COVID-19 pandemic underlined, when living and working conditions compounded their risk of exposure to the virus (https://www.thelancet.com/journals/laninf/article/PIIS1473-3099(21)00410-2/fulltext).

Third, Member States committed to creating inclusive environments that promote public health, social inclusion, and well-being—acknowledging that refugees’ and migrants’ health depends on multiple factors beyond the health sector. Formal access to services is important, but this alone will not improve refugee and migrant health. Underlying social, economic, and environmental determinants need to be reckoned with. Integration and social cohesion, to take just one example, are essential to building inclusive, healthy environments in which refugees and migrants thrive alongside host populations.

Fourth, Member States pledged to strengthen migration-related health governance and policymaking. Safe, responsible and dignified migration is only possible with good governance, driven by evidence and data. As an example, in many countries across the WHO European Region, migrants make up a significant proportion of the health sector. In London and Brussels, for instance, around half of all doctors and nurses are migrants (https://www.oecd.org/coronavirus/policy-responses/Covid-19-and-key-workers-what-role-do-migrants-play-in-your-region-42847cb9/). With proper governance, migration can be utilized to address shortages of skilled labour, while also ensuring brain drain does not harm other societies. In short, migration can pose challenges but also offer enormous opportunities.

Fifth, Member States committed to exploring innovative ways of working and developing new partnerships. This action pillar seems straightforward; clearly, countries and stakeholders need to work together. However, as myriad factors outside the health sector influence refugees’ and migrants’ health, the importance of inter-sectoral and whole-of-route approaches cannot be underestimated. From ministries of interior to social services, interventions and programmes that fall under a range of government entities need to be coordinated and co-led to build genuinely inclusive systems.

WHO/Europe is working closely with its Member States to implement the action plan rolled out last year by demonstrating health leadership, sharing the latest international evidence, and offering tailored advice to countries. In April 2024, the Regional Office co-hosted the first Knowledge Forum on Refugee and Migrant Health to provide resources and tools for countries to help make their commitment to health for all, including refugees and migrants, a reality (https://www.who.int/europe/news-room/events/item/2024/04/16/default-calendar/knowledge-forum-on-refugee-and-migrant-health).

As well, we are collaborating with our counterparts in the WHO African and Eastern Mediterranean Regions to better integrate our individual efforts on refugee and migrant health, given the migration routes and pathways that stretch across geographies in our interconnected world.

One’s health should not be determined by where you come from. The right to health is a human right for all, not just a privilege of the few. It is the right of the 1 in 30 people globally who live outside their country of origin. For Member States in WHO European Region that have reached agreement on paper, words must now be followed by action.

## Sailing the waves of European public health: exploring a sea of innovation

MexiaRicardoChair of the 17th EPH Conference, Lisbon 2024BarnhoornFloris
Deputy Director EPH Conference

**17th European Public Health Conference, Lisbon, Portugal**

**Pre-Conferences 11–12 November**

**Main Conference 13–15 November**

**Sailing the Waves of European Public Health: Exploring a Sea of Innovation**

The 17th EPH Conference in Lisbon, Portugal, is themed ‘Sailing the waves of European public health: exploring a sea of innovation’. Just as explorers once set out from Lisbon to discover new worlds, we as public health professionals are embarking on a journey of innovation and discovery in our own field. The conference includes an extensive programme, with 16 tracks focusing on the conference themes and key areas of public health. The conference also offers numerous networking opportunities and opportunities to meet colleagues from Europe and beyond. As usual, there is an extensive programme of pre-conferences on the days before the main conference.

### Pre-conference programme

We are excited to announce the ‘*Conferência de Saúde Pública da Lusofonia*’, on 11 and 12 November 2024, specifically targeting the global Lusophone community. This initiative recognizes and celebrates the unique cultural ties among Portuguese-speaking countries, aiming to foster a collaborative environment for public health innovation and discussion. Thematic areas include National Health Systems, Training and Development of the Health Workforce, Health Information and Communication, Monitoring of Sustainable Development Goals, Public Health Emergency Preparedness, Research and Bioethics in Health. These themes will be addressed in plenary sessions and round tables, spread out over the 2-day conference. The conference language will be Portuguese. Simultaneous translation into English will be provided. Detailed information **here**. The Lusophone Public Health Conference is organized by EPH Conference, the European Public Health Association (EUPHA), the Portuguese Association for Public Health (APPSP), and the National Association of Public Health Doctors (ANMSP).

More pre-conferences are organized on Tuesday, 12 November. Thematic fields include cancer prevention; health literacy; European Health Data Space; Artificial Intelligence; digital health; strategic foresight methodology; behavioural and cultural insights; equitable vaccination; social security, work and health; prevention and preparedness; syndromic surveillance systems; enhancing your writing skills; and future health scenarios. Detailed information **here**.

### Plenary programme

The theme of this year’s EPH Conference ‘**Sailing the waves of European public health: exploring a sea of innovation**’, and the conference subthemes are dealt with in the plenary sessions. The focus of our exploratory journey will be on:

- Artificial Intelligence in Public Health: exploring how AI is transforming disease surveillance, healthcare delivery, and decision-making
- One Health: discussing the interconnectedness of human, animal, and environmental health, how to strengthen public health strategies and mitigate emerging threats
- Social Marketing in Public Health: presenting innovative campaigns, digital and community engagement techniques to promote healthier behaviours
- Health Inequalities: looking at innovative strategies that can be employed to reduce disparities and ensure equitable access to health care
- Global Health: how can European public health efforts contribute to global health solutions

The themes will be dealt with in our plenary sessions, organized by EUPHA, EuroHealthNet, European Health Management Association (EHMA), ASPHER, European Observatory on Health Systems and Policies, European Centre for Disease Prevention and Control (ECDC), European Commission, and WHO Regional Office for Europe. Read more **here**.

The 17th EPH Conference in Lisbon invites public health professionals and students to navigate the vast ocean of possibilities within our field, charting a course towards a future where innovation and collaboration lead the way in addressing the complex health challenges of our time.

Keep updated on all the details at the conference website: www.ephconference.eu

See you in Lisbon!

Ricardo Mexia, Chair of the 17th EPH Conference, Lisbon 2024

